# Does soil history decline in influencing the structure of bacterial communities of *Brassica napus* host plants across different growth stages?

**DOI:** 10.1093/ismeco/ycae019

**Published:** 2024-01-31

**Authors:** Andrew J C Blakney, Marc St-Arnaud, Mohamed Hijri

**Affiliations:** Institut de recherche en biologie végétale, Département de Sciences Biologiques, Université de Montréal and Jardin botanique de Montréal, Montréal, Québec, H1X 2B2, Canada; Present address: Department of Physical and Environmental Sciences, University of Toronto, Scarborough, Ontario, M1C 1A4, Canada; Institut de recherche en biologie végétale, Département de Sciences Biologiques, Université de Montréal and Jardin botanique de Montréal, Montréal, Québec, H1X 2B2, Canada; Institut de recherche en biologie végétale, Département de Sciences Biologiques, Université de Montréal and Jardin botanique de Montréal, Montréal, Québec, H1X 2B2, Canada; African Genome Center, Mohammed VI Polytechnic University (UM6P), Lot 660, Hay Moulay Rachid, Ben Guerir 43150, Morocco

**Keywords:** Brassica napus, bacterial communities, soil history, growth stages, rhizosphere

## Abstract

Soil history has been shown to condition future rhizosphere microbial communities. However, previous experiments have also illustrated that mature, adult plants can “re-write,” or mask, different soil histories through host plant–soil community feedbacks. This leaves a knowledge gap concerning how soil history influences bacterial community structure across different growth stages. Thus, here we tested the hypothesis that previously established soil histories will decrease in influencing the structure of *Brassica napus* bacterial communities over the growing season. We used an on-going agricultural field experiment to establish three different soil histories, plots of monocrop canola (*B. napus*), or rotations of wheat-canola, or pea-barley-canola. During the following season, we repeatedly sampled the surrounding bulk soil, rhizosphere, and roots of the *B. napus* hosts at different growth stages—the initial seeding conditions, seedling, rosette, bolting, and flower—from all three soil history plots. We compared composition and diversity of the *B. napus* soil bacterial communities, as estimated using 16S rRNA gene metabarcoding, to identify any changes associated with soil history and growth stages. We found that soil history remained significant across each growth stage in structuring the bacterial bulk soil and rhizosphere communities, but not the bacterial root communities. This suggests that the host plant’s capacity to “re-write” different soil histories may be quite limited as key components that constitute the soil history’s identity remain present, such that the previously established soil history continues to impact the bacterial rhizosphere communities, but not the root communities. For agriculture, this highlights how previously established soil histories persist and may have important long-term consequences on future plant–microbe communities, including bacteria.

## Introduction

Plants have never existed without microorganisms, hence the critical impact microbes have on plant metabolism, growth, and survival [1–3]. For instance, soil microbes increase access to nutrients [4–6], temper the impacts of environmental changes [7], or stress [8, 9], protect against infection from pathogen [10, 11], and cue each plant developmental stage [7, 12, 13]. For example, microbial communities can shift the timing or transition to different growth stages through nutritional or phytohormone pathways [13]. The ubiquitous soil microbial communities help integrate these diverse signals and modulate the plant’s responses [9, 14]. Such a long-term relationship, where plants interact with similar cohorts of microbial traits across generations, highlights a host plants capacity to tailor the structure of their bacterial communities in response to variable conditions and the plant’s needs through time [6, 15–17]. As the soil is the source of the majority of plant-associated microbes [18], host plants have the capacity to alter the local soil chemistry through two concurrent processes; first, the host plant’s growth, development, and homeostasis are determined by its capacity to uptake nutrients from the soil, which will influence the soil chemistry [19]. Second, through rhizodeposition, the host plant can vary the quantity and array of compounds released into the rhizosphere as required, i.e. the soil influenced by the plant host. Thus, through these processes, plants change the local soil chemistry, thereby tailoring the structure of their bacterial communities as appropriate [6, 15–17, 20].

In turn, rhizosphere microbial communities will continuously incorporate the various biotic and abiotic factors and establish a reciprocal feedback process for the benefit of the current plant–microbial community [21–23]. This above-ground–below-ground reciprocal feedback [9] will go on to impact future microbial generations and their composition [24–27]. Thus, physicochemical information from one rhizosphere microbial community is transmitted through time to impact subsequent plant–microbial generations, i.e. that the soil history, also referred to as soil legacy, of previous rhizosphere microbial communities’ condition future ones [25, 28, 29]. Future host plants can then alter the soil bacterial communities for their own purposes, in a broadly phylogenetic-dependent manner [27, 30]. However, several questions remain surrounding the duration of different soil histories, what their impact on future plant–microbial communities may be, or how quickly rhizosphere microbial community feedback may alter or “re-write” different soil histories.

Furthermore, soil history will dictate how future rhizosphere microbial communities assemble by establishing the biotic and abiotic context of the soil environment, but also through priority effects [31–33]. As a new plant host develops, not only could its potential microbial community be constrained according to the soil history, but also according to the composition at an earlier growth stage. For example, more recently increased attention has been paid to seed [34–38] and flower microbial communities [39–41]. These experiments focus on how microbial communities are vertically transmitted, subsequently establish new communities, and cue germination [13], but are compositionally constrained by previous growth stages [13]. In fact, microbes may be critical at early life stages; seed and seedling growth stages are precarious periods where the plant is most vulnerable to environmental stress and infection from phytopathogens [21, 42]. Unfortunately, however, most experiments testing soil history have only focused on the microbial communities of adult plants [27, 29, 30, 43].

Our previous experiment illustrated how different soil histories could be “re-written” by host plant–soil microbial community feedback (PSF), or persist for up to a year [30, 43]. However, since we only sampled adult host plants, we were unable to explore the strength or variation of different soil histories on the microbial communities throughout the growing season. Consequently, not incorporating temporal sampling has explicitly ignored the different roles of time on the assembly of microbial communities [44–46]. This has left a severe knowledge gap of how soil history impacts microbial communities at different growth stages of the host plant, as well as how these communities develop throughout the growing season [42]. Thus, experiments are needed to test the influence and dynamics of soil microbial communities at a variety of growth stages.

In this study, we investigated how different soil histories impacted soil bacterial communities at five developmental growth stages throughout the season. We partnered with an on-going agricultural field experiment, as crop rotations and their agricultural inputs provide a ready-made model for how the previously established soil history of different crops impacts future plant–bacterial communities [22, 23]. This helps address the lack of field experiments in studying soil history [47]. Here, the three established soil histories were rotated plots of monocrop canola (*Brassica napus*), wheat-canola (WC), and pea-barley-canola (PBC). During the following season, we repeatedly sampled *B. napus* and surrounding soil at different growth stages—the initial seeding conditions, seedling, rosette, bolting, and flower—from all three soil history plots. This design permitted us to test the hypothesis that the previously established soil histories—monocrop, WC, PBC, and their respective microbial communities and agricultural treatments—would decrease in influencing the structure of the *B. napus* bacterial rhizosphere communities over the growing season, as the host’s PSF reconstructs the existing soil history. To help us identify the competing influence of soil history and growth stages, we compared the rhizosphere communities to their corresponding bulk soil and root communities at each growth stage. Bulk soil communities were a reference point to help account for the accumulative impact of time, the random ecological effects that can occur, and the different soil histories on the soil bacterial communities throughout the growing season, but without the influence of the plant hosts at each growth stage. Conversely, the bacterial root communities were a reference point to account for the dominant influence of the host through time, but without the impact of the soil history, as illustrated by our previous experiment [30].

We made four predictions that will help illustrate support for our “fading soil history through time” hypothesis: (i) that the bacterial bulk soil communities will remain stable and continue to be primarily structured by their soil histories throughout the experiment, as they should not, by definition, be impacted by a plant host, (ii) conversely, the bacterial root communities will be primarily structured by the different growth stages and shift accordingly, regardless of the soil history, (iii) the bacterial rhizosphere communities will be primarily structured by their soil histories at the seedling growth stage and resemble their cognate bulk soil communities, and (iv) as a result of the declining influence of soil history, at the flower stage, the bacterial rhizosphere communities will be primarily structured by the on-going PSF of the current *B. napus* host plant, and so ought to be more similar to one another and divergent from their cognate bulk soil communities. To test our “fading soil history through time” hypothesis, we estimated the bacterial communities from the bulk soil, rhizosphere, and roots of *B. napus* plants throughout the growing season using a 16S rRNA gene metabarcoding approach to infer amplicon sequence variants (ASVs). We compared the taxonomic composition and diversity of the bacterial communities to identify any changes associated with different soil histories and growth stages on the *B. napus* soil bacterial communities.

## Materials and methods

### Site and experimental design

A long-term crop rotation field experiment is on-going at the experimental farm of Agriculture and Agri-Food Canada’s Research and Development Centre, in Lacombe, Alberta (52°28′06″N, 113°44′13″W). The site is located in the semi-arid region of the Canadian Prairies; according to the weather station at the research farm, the 2019 growing season (May, June, and July) had 197.8 mm of precipitation; compared with the 30-year average [1981–2010] of 216.3 mm (Table S1). The daily temperature average for the 2019 season was 12.6°C, while the 30-year average was 14.5°C (Table S1). The farm has a loam texture (46% sand, 33% silt, and 21% clay) and has been well-described previously [48].

The experiment reported here was derived from the two-phase cropping sequence between the 2018 and 2019 growing seasons. The experimental design was a split-plot replicated in four complete blocks (Fig. S1B). For the 2018 Conditioning Phase, we selected three soil history treatments that consisted of (i) monocrop canola (*B. napus* L., cv. L252LL), (ii) a 2-year crop rotation between spring wheat (*Triticum aestivum* cv. AAC Brandon) and *B. napus* (WC), and (iii) a 3-year rotation between pea (*Pisum sativum* L. cv AAC Lacombe), barley (*Hordeum vulgare* cv. Canmore), and *B. napus* (PBC; Fig. S1). Thus, the 2018 Conditioning Phase established soil histories composed of either canola, wheat, or barley, plus their respective management plans described below [30, 48]. In the 2019 test phase, the 12 Conditioning Phase plots were all seeded with *B. napus*. The test phase evaluated the on-going PSF from the *B. napus* host, the soil bacterial community, the on-going management plans, and previous soil history [30, 43].

### Crop management and sampling

Crops were grown and maintained according to standard management practices, as previously described [48]. Briefly, in the 2018 Conditioning Phase, a pre-seed “burn off” herbicide treatment using glyphosate (Roundup, 900-g acid equivalent per hectare, a.e. ha^−1^) and bromoxynil (Pardner, 280–330 g active ingredient per hectare, a.i. ha^−1^) was applied to all plots to ensure a clean starting field prior to seeding. The herbicide Liberty was applied to *B. napus*, while Pixxaro A and B with Axial were applied to wheat and barley plots, for in-season weed control; fungicides were only applied as needed. Soil tests were used to determine the rates of in-season nitrogen, phosphorus, and potassium application. Crops were harvested between late August and early October, depending on the crop. During the subsequent 2019 test phase, *B. napus* plant hosts were subjected to the same standard management practices as the Conditioning Phase, including pre-seed “burn off,” in-season herbicide, and fungicide treatments as needed, and fertilized as recommended by soil tests.

Test phase *B. napus* plants were sampled at specific growth stages, seed, seedling, rosette, bolting, and flower, as described by the Canola Council of Canada [48]. First, at the seed stage (GS00, 10th May 2019), we took a 25-mL sample of the *B. napus* seeds to be seeded, as well an equivalent amount of soil from each plot. At the seedling stage, post-emergence, when only the cotyledons were visible (GS10, 27th May 2019), five seedlings and their accompanying soil were pooled together for each sample. Composite samples were taken only at the seedling stage in order to have enough root material for our subsequent DNA extractions. At the rosette, bolting, and flower stages, individual plants and their associated soil were harvested from each plot. The rosette stage (GS19, 18th June 2019) was harvested when nine leaves were visible, followed by the bolting stage (GS34, 2nd July 2019) when a 20-cm stem was present. Finally, the flower stage (GS65, 15th July 2019) was harvested when 50% of the flowers on the raceme were open [49].

At each growth stage, we sampled three compartments: bulk soil, rhizosphere, and root. Within each plot, bulk soil was sampled from between the seeded rows, at least 10 cm from any seeds, or plants. Note that at the seed stage, the only material collected was bulk soil and the seeds. In the field, each plant had its aerial portions removed, and its roots and accompanying soil stored in coolers on ice. Based on the sampling strategy, in this study, we define the bulk soil microbiome as the soil bacterial community not influenced by the resident host plant, the rhizosphere microbiome as the bacterial community in the soil in close contact with the roots [29], and the root microbiome as the bacterial community attached to, and within, the roots [26]. We accounted for the use of the various agricultural treatments in the downstream amplicon data by considering each plant sample and their total complement of particular agricultural treatments as a unit [30] (see Supplementary Materials). In the field, all samples were kept on ice in coolers, then stored in the lab at −80°C before being shipped to Université de Montréal’s Biodiversity Centre, Montréal (QC, Canada) on dry ice for further processing [50, 51].

### DNA extraction from the test phase *B. napus* samples

Total DNA was extracted from all compartments (bulk soil, seeds, rhizosphere, and roots) of the Test Phase field trial samples (see Supplementary Materials for details). Roots were first sieved out of the total soil sample and gently scraped it off using sterilized utensils into fresh collection trays. Seeds and root samples were ground separately in liquid nitrogen via sterile mortar and pestles (Fig. S2). For bulk soil and rhizosphere samples, ~500 mg was used for the NucleoSpin Soil gDNA Extraction Kit (Macherey-Nagel, Germany), while ~130 mg of seeds and roots were used with the DNeasy Plant DNA Extraction Kit (Qiagen, Germany) [30, 43, 51]. We failed to extract DNA from nine of the seedling root samples due to a lack of material; those samples were subsequently excluded hereafter (Fig. S2).

To estimate the composition of the bacterial communities in the bulk soil, seed, rhizosphere, and roots from the *B. napus* growth stages, extracted DNA from all samples were used to prepare 16S rRNA gene amplicon libraries using Illumina’s MiSeq platform (Génome Québec, Montréal) [30, 43, 51, 52]. ASVs were then identified from the raw 11 010 728 MiSeq reads [53]. The quality of the data was assessed using the included controls (Fig. S4), including a commercially available 16S rRNA mock community, of known composition (Table S2) rarefaction curves confirmed that we captured the majority of the bacterial communities in both the bulk soil, seed, rhizosphere, and roots (Fig. S5). estimated the absolute abundance, or size, of the bacterial communities in each DNA sample by qPCR [30, 43, 54] (see the Supplementary Materials for details Table S3, Fig. S6).

### α-Diversity of the test phase *B. napus* bacterial communities

In order to estimate the coverage of the bacterial domain of life, we calculated Faith’s phylogenetic diversity as an α-diversity index from the *B. napus* samples using the pd function from the picante package (sum of all branch lengths separating taxa in a community) [55]. To refine our understanding of the abundance and composition of the *B. napus* bacterial communities, we used two complementary methods to identify taxa specific to growth stages and soil history. First, taxa cluster maps were used to calculate the differential abundance of ASVs between experimental groups [56]. Second, indicator species analysis was used to detect ASVs that were preferentially abundant in pre-defined environmental groups (compartments, growth stages, soil history) [57]. Given the large number of taxa in our study, it was not practical to view taxa clusters as matrices below class, whereas indicator species analysis pinpoints specific ASVs of interest. Please refer to the Supplementary Materials for details, including the statistical analysis.

### β-Diversity of the test phase *B. napus* bacterial communities

To test for significant differences between the *B. napus* bacterial communities from different growth stages and soil histories, we used the non-parametric permutational multivariate ANOVA (PERMANOVA; Table S4). The PERMANOVA was calculated using the adonis function in the vegan package [58], with 9999 permutations, and the experimental blocks were included as “strata.” This was complemented with a PERMANOVA for each compartment (bulk soil, rhizosphere, and roots) that specifically tested growth stages and soil histories as experimental factors, and used a weighted Unifrac distance matrix [59, 60] calculated using the distance function in phyloseq [61] (see Supplementary Materials for details).

Distance-based redundancy analyses (RDAs), using UniFrac distances weighted by absolute abundance, were used to quantify the amount of variation described by each experimental factors (constrained by: soil history or growth stages) in the bacterial communities from the bulk soil, rhizosphere, or roots (i.e. how much of the phylogenetic change between communities was due to the compartment, soil history, or growth stages, where communities with similar phylogenetic composition appear closer together; [57]). Model accuracy was assessed with an adjusted *R*^2^ value and tested for significance using an ANOVA [62].

## Results

### Bulk soil and rhizosphere samples were more similar than to root or seed samples

Illumina’s MiSeq produced 11 010 728 raw reads for the whole dataset, which were then processed through DADA2 [53, 63], where we retained 2 770 390 reads from all the experimental samples, which inferred a total of 33 392 ASVs (Table 1 and Fig. S3; see Supplementary Materials for details). Globally, we found that the bacterial communities from the bulk soil, seeds, rhizosphere, and roots were significantly different (PERM *R*^2^ = 0 .60135, *P* < 0.001). β-Diversity analysis highlighted the difference between the root and seed communities from the bulk soil and rhizosphere communities (Fig. S8A). These differences were further reflected by significantly different levels of phylogenetic diversity, where the bulk soil and rhizosphere communities remained the most diverse throughout the growing season (*P* < 0.001, Fig. S7). Comparatively, the root and seed communities remained consistently less diverse (Fig. S7). Indicator species analysis did not identify any specific ASVs according to compartment, growth stage, nor soil history.

**Table 1:** The bacterial bulk soil and rhizosphere communities had more unique ASVs than the root communities at each growth stage of their host plant *B. napus*, harvested throughout the 2019 growing season in Lacombe, Alberta.

Growth stage*^a^*	Compartment*^b^*	16S rRNA reads*^c^*	ASV occurrence*^d^*	16S rRNA gene copies*^e^*
Seed	Bulk (*n* = 12)	21 213 ± 1842	1497 ± 128	2 360 322 ± 1 610 500
	Seed (*n* = 1)	7	6	2 251 078
Seedling	Bulk (*n* = 12)	22 886 ± 1516	1526 ± 72	1 224 464 ± 1 083 033
	Rhizosphere (*n* = 12)	19 817 ± 3941	1355 ± 143	6 722 767 ± 1 929 973
	Root (*n* = 3)	2519 ± 117	432 ± 28	1 153 665 ± 738 663
Rosette	Bulk (*n* = 12)	23 020 ± 4472	1448 ± 128	1 415 797 ± 1 088 160
	Rhizosphere (*n* = 12)	19 984 ± 7475	1295 ± 444	2 131 129 ± 833 941
	Root (*n* = 12)	3058 ± 1099	361 S ± 67	13 105 921 ± 5 757 254
Bolting	Bulk (*n* = 12)	26 811 ± 6475	1567 ± 209	3 266 621 ± 1 896 126
	Rhizosphere (*n* = 12)	29 286 ± 6090	1611 ± 219	2 305 561 ± 1 249 093
	Root (*n* = 12)	2502 ± 3362	231 ± 116	8 711 539 ± 4 380 029
Flowering	Bulk (*n* = 12)	28 704 ± 6599	1649 ± 150	3 049 801 ± 3 738 808
	Rhizosphere (*n* = 12)	23 263 ± 4147	1552 ± 132	10 044 431 ± 5 541 865
	Root (*n* = 12)	4353 ± 3784	352 ± 105	3 159 861 ± 1 327 309

a Test phase growth stages.

b Presented with the number of samples (*n*) retained.

c Values are presented as mean ± SD.

d Values are presented as mean ± SD.

e Estimated by qPCR as the number of 16S rRNA gene copies; values are presented as mean ± SD.

### Bacterial bulk soil communities impacted more by soil history than growth stages

**Figure 1:**
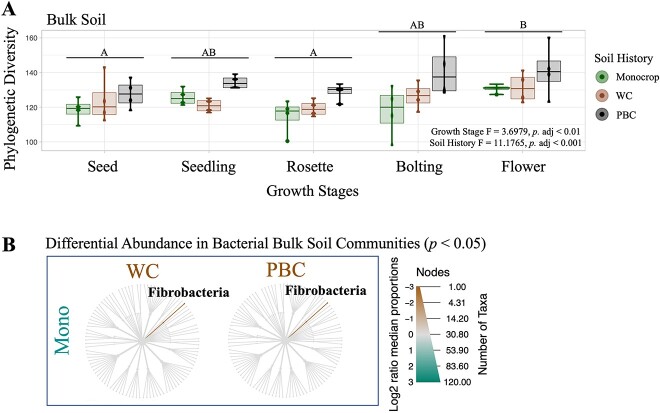
Bacterial communities identified from bulk soil samples were largely stable in phylogenetic diversity (A) and taxa (B) across different growth stages and soil histories from samples harvested throughout the 2019 *B. napus* growing season in Lacombe, Alberta; (A) diversity across growth stages and soil histories was tested with a multi-factor ANOVA, after which statistically significant groups were identified using Tukey’s *post hoc* test; (B) taxa that were significantly more abundant, as determined using the non-parametric Kruskal–Wallis test, followed by the *post hoc* pairwise Wilcox test, are highlighted brown or green, following the labels for each compared host.

To test our hypothesis that the previously established soil histories would decrease in influencing the structure of the *B. napus* bacterial rhizosphere communities over the growing season, we first sought to establish the dynamics through time of the corresponding bulk soil communities. We did this to help determine the impact of the different soil histories through time on the bacterial communities, but without the influence of the plant host’s growth stages. Soil history and growth stages (i.e. time in the context of the bulk soil) were both significant in the bulk soil communities, though the interaction was not (PERM *R*^2^ = 0.08770, *P* < 0.001; *R*^2^ = 0.08596, *P* < 0.016, respectively; Table 2). Phylogenetic diversity remained stable across growing season, except at the flower stage where diversity was significantly higher (A vs B, *P*. adj < 0.01; Fig. 1A). Furthermore, bulk soil communities with a soil history of PBC had significantly higher phylogenetic diversity at each growth stage time point, compared with the communities from monocrop or WC plots (*P*. adj < 0.001; Fig. 1A). Monocrop bulk soil communities were also globally depleted in *Fibrobacteria*, compared with WC or PBC bulk soils (*P* < 0.05; Fig. 1B). The bacterial bulk soil communities were enriched in class ABY1 (phylum *Patescibacteria*) at the rosette stage (*P* < 0.05; Fig. S9B), and in class *Rhodothermia* (phylum *Bacteroidota*) at the bolting stage (*P* < 0.05; Fig. S9C), when compared with their cognate rhizosphere communities.

**Table 2:** PERMANOVA identified growth stage and soil history as significant experimental factors for the bacterial bulk soil and rhizosphere communities harvested in 2019 from *B. napus* in Lacombe, Alberta, and PERMANOVA was calculated using a weighted Unifrac distance matrix, with 9999 permutations.

Experimental factor	Test phase compartments^a^								
	Bulk soil			Rhizosphere			Roots		
	*F* model	*R* ^2^	Pr (>*F*)	*F* model	*R* ^2^	Pr (>*F*)	*F* model	*R* ^2^	Pr (>*F*)
Growth stage^b^	1.31843	0.08596	**0.016**	2.04117	0.12043	**0.001**	2.33385	0.16454	**0.006**
Soil history^c^	2.69030	0.08770	**0.001**	2.73273	0.10749	**0.001**	1.40096	0.06585	0.163
Soil history ~ growth stage	0.71201	0.09285	0.928	0.70968	0.08374	0.812	0.95793	0.13508	0.548

a Values in bold indicate significant factors or interactions.

b Test phase growth stages: seed, seedling, rosette, bolting, or flower.

c Soil history established the previous year: monocrop canola (*B. napus*), WC rotation, or PBC rotation.

**Figure 2:**
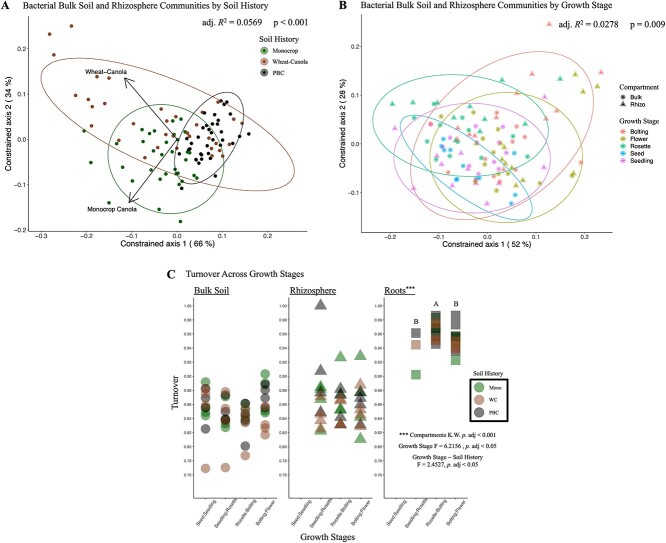
Bacterial communities identified from the bulk soil and rhizosphere of *B. napus* host plants were more phylogenetically similar in agricultural plots with PBC soil history (A) and at early growth stages (B); distance-based redundancy analysis, using UniFrac distances weighted by absolute abundance, quantified how the experimental factors (A, constrained by soil history) and (B, constrained by growth stages) structured the bacterial communities, where those with similar phylogenetic composition appear closer together; (C) β-diversity of bacterial communities were dominated by turnover across each growth stage of *B. napus*, particularly in the roots, and significant differences in turnover among compartments were identified using the non-parametric Kruskal–Wallis test, followed by the *post hoc* pairwise Wilcox test. Within a compartment, a multi-factor ANOVA was used to test for significance among growth stages and soil histories, after which statistically significant groups were identified using Tukey’s *post hoc* test. Abbreviations: Kruskal–Wallis (K.W.).

RDA also showed that the bulk soil communities with PBC soil history were more phylogenetically consistent to each other across the growing season than to either the monocrop, or WC, as all the PBC soil communities remained more clustered (adj. *R*^2^ = 0.0569, *P* < 0.001; Fig. 2A). We also observed that the bulk soil communities at the seed and seedling communities tended to be more phylogenetically similar, while the remaining time points (rosette, bolting, and flower) were more diverse (adj. *R*^2^ = 0.0278, *P* = 0.009; Fig. 2B). Partitioning the β-diversity illustrated that the bacterial bulk soil communities were dominated by turnover, or species replacement, throughout the growing season, regardless of their different soil histories, though no transition between time points was significant (Fig. 2C).

### Bacterial root communities were only impacted by growth stage, and not soil history

**Figure 3:**
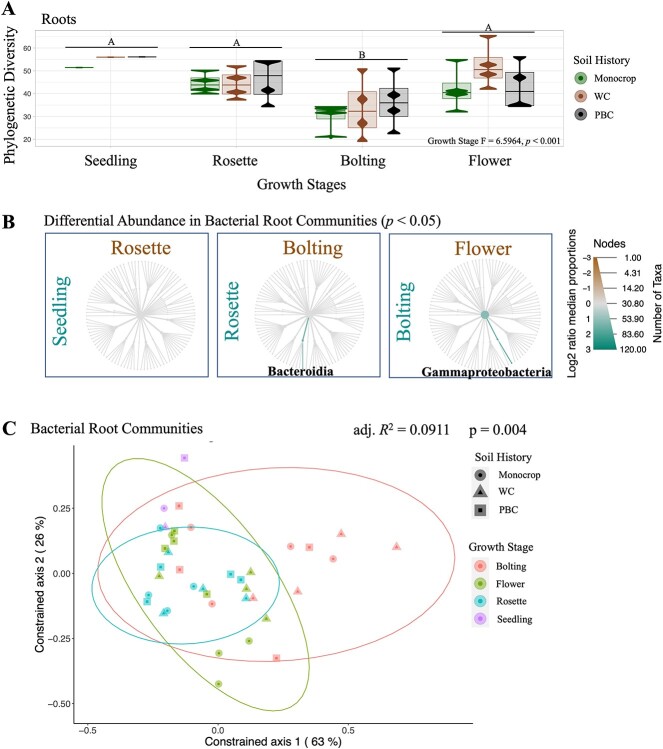
Bacterial root communities were stable in phylogenetic diversity (A) and taxa (B) across different growth stages and soil histories throughout the 2019 *B. napus* growing season in Lacombe, Alberta, and were more phylogenetically similar to each other at early growth stages (seedling, purple, and rosette, blue) compared with later growth stages (C), and (A) phylogenetic diversity across growth stages was tested with a multi-factor ANOVA, after which statistically significant groups were identified using Tukey’s *post hoc* test. (B) Taxa that were significantly more abundant, as determined using the non-parametric Kruskal–Wallis test, followed by the *post hoc* pairwise Wilcox test, are highlighted brown or green, following the labels for each compared host, and (C) distance-based redundancy analysis, using UniFrac distances weighted by absolute abundance, quantified how the experimental factors (constrained by growth stages) structured the bacterial communities, where those with similar phylogenetic composition appear closer together. Abbreviations: wheat-canola (WC), pea-barley-canola (PBC).

Next, to establish the influence of the plant-induced growth stages, but with minimal impact from the different soil histories, we analyzed the dynamics of the bacterial root communities across the different growth stages. Here, we found that only growth stages were significant in structuring root communities (PERM *R*^2^ = 0.16454, *P* < 0.006; Table 2), as opposed to the bulk soil communities where both the time points and soil history were significant. RDA further illustrated the significant impact of growth stages on the root communities, with no impact of soil history (adj. *R*^2^ = 0.0569, *P* < 0.001; Fig. 3C). We also observed that the root communities were more phylogenetically consistent at the seedling stage and became more variable at each subsequent growth stage (adj. *R*^2^ = 0.0911, *P* < 0.004; Fig. 3C). Finally, partitioning the β-diversity also highlighted the importance of growth stages in the roots, as turnover was significantly higher during transition from rosette to bolting in the roots (*P.* adj < 0.05; Fig. 2C). Bacterial community turnover was also significantly higher in the roots than in the bulk soil, or rhizosphere (*P*. adj < 0.001; Fig. 2C).

In the root communities, phylogenetic diversity was low compared with the bulk soil and rhizosphere communities (Fig. S7). We did not detect any impact of the different soil histories on the phylogenetic diversity, nor differential abundances of taxa, in the root communities at any growth stage. These communities were very stable across growth stages, except at the bolting stage, where phylogenetic diversity was significantly lower compared with the other growth stages (*P*. adj < 0.01; Fig. 3A). The root communities were significantly depleted in a wide variety of bacterial taxa at each growth stage when compared with the rhizosphere (*P* < 0.05; Fig. S9C). Nevertheless, the roots were also significantly enriched in specific taxa, compared with the rhizosphere (Fig. S9). First, at the rosette stage, the most prominently enriched taxa in the roots were in the *Verrucomicrobiae*, *Actinobacteria*, *Proteobacteria*, and *Bacterodia* (*P* < 0.05; Fig. S9B). Second, at the bolting stage, root communities were enriched in the *Gammaproteobacteria* and *Bacterodia* (*P* < 0.05; Fig. S9C). We also detected two enriched taxa in the roots when compared among themselves at different growth stages; *Bacterodia* were enriched at the rosette stage, compared with the bolting stage, while *Gammaproteobacteria* were enriched at the bolting stage, compared with the flower stage (*P* < .05; Fig. 3B).

### Rhizosphere communities were impacted by growth stages and soil history

Finally, we compared how the dynamics of the bacterial rhizosphere communities were influenced by growth stages and soil history. Similar to bulk soil communities, soil history and growth stages were also both significant for the rhizosphere communities, while the interaction was not (PERM *R*^2^ = 0.10749, *P* < 0.001; *R*^2^ = 0.12043, *P* <0 .016, respectively; Table 2). RDA illustrated that the rhizosphere communities remained phylogenetically similar with the bulk soil communities across the growing season, being most similar at the early growth stages (seed and seedling) versus later growth stages (rosette, bolting, and flower; Fig. 2B, adj. *R*^2^ =0 .0278, *P* = 0.009). Also such as the bulk soil communities, the PBC communities were more phylogenetically consistent to each other, regardless of growth stage, than to either the monocrop, or WC (Fig. 2A, adj. *R*^2^ = 0.0569, *P* < 0.001).

**Figure 4:**
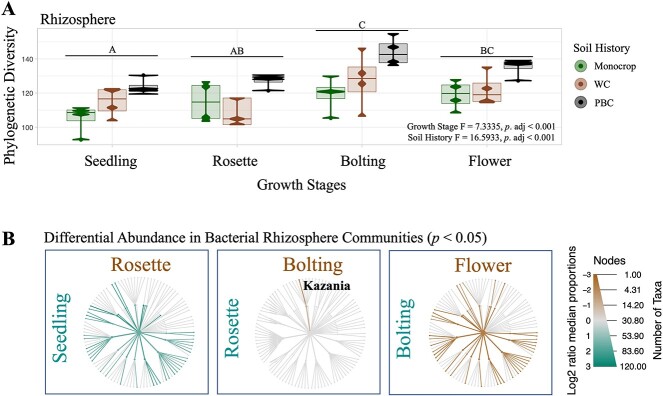
Bacterial rhizosphere communities varied significantly in phylogenetic diversity (A) and taxa (B) between growth stages of their *B. napus* plant host, harvested throughout the 2019 growing season in Lacombe, Alberta; (A) diversity across growth stages and soil histories was tested with a multi-factor ANOVA, after which statistically significant groups were identified using Tukey’s *post hoc* test, and (B) taxa that were significantly more abundant, as determined using the non-parametric Kruskal–Wallis test, followed by the *post hoc* pairwise Wilcox test, are highlighted brown or green, following the labels for each compared host. Abbreviations: wheat-canola (WC), pea-barley-canola (PBC).

Rhizosphere communities with PBC soil history also had significantly more phylogenetic diversity at each growth stage, than the rhizosphere communities from either the monocrop or WC soil histories (*P*. adj < 0.001; Fig. 4A). However, phylogenetic diversity generally increased across growth stages regardless of previous soil histories, such that the bolting and flower rhizosphere communities were more diverse than those from the seedling and rosette growth stages (*P*. adj <0 .001; Fig. 4A). A similar trend was observed in the β-diversity, such that rhizosphere communities had more variation at the rosette, bolting, and flower stages compared with communities in the bulk soil (Fig. 2B). The β-diversity of the rhizosphere communities was also driven by turnover across each growth stage and was unaffected by different soil histories, similar to the bulk soil communities (Fig. 2C).

We observed a number of significant changes to taxonomic abundances in the rhizosphere, compared with the bulk soil and root communities, at several growth stages (*P* < 0.05; Fig. S9), with the most prominent and widespread enrichment being during the seedling and flower stages (*P* < 0.05; Fig. 4B). The rhizosphere communities were largely stable among themselves between the rosette and bolting stages in terms of different taxa abundance, except for an enrichment in *Kazania* bacteria (phylum *Desulfobacterota*; Fig. 4B). However, they were noticeably enriched in a number of taxa compared with the roots at the rosette and bolting stages (Fig. S9B and C). There were no ASVs that were significantly enriched, or depleted, in the rhizosphere according to growth stages or soil history.

## Discussion

Soil history implies that some physicochemical signal, or information, is transmitted through time to condition the assembly of future rhizosphere microbial communities [25, 28, 29]. Although our previous work has shown this can be true [30, 43], the influence or longevity of soil history on bacterial community structure across growth stages is not clear. In this study, we tested how previously established soil histories endured across growth stages throughout a growing season. We hypothesized that previously established soil histories will decrease in influencing the structure of *B. napus* bacterial rhizosphere communities over the growing season. We took advantage of an agricultural field experiment to bridge the gap between controlled greenhouse conditions and experiments in “natural” environments, as such studies are currently lacking to understand bacterial temporal dynamics [47, 64, 65]. We sampled bulk soil, rhizosphere, and roots successively throughout the *B. napus* growing season from plots with different soil histories and used 16S rRNA gene metabarcoding to identify the bacterial communities. Contrary to our hypothesis, the weight of evidence suggests that the different soil histories did not decline in influence, but rather continued to have a significant impact on the bacterial rhizosphere communities throughout the growing season.

These communities are initially established at the confluence of the soil environment and the immediate space the plant host may be able to influence [29, 66]. Given that the new *B. napus* host would be mere days emerging from its seed into a new soil environment with an established soil history [66], and minimally able to exert much influence on the surrounding soil through PSF [42], we predicted that at the early seedling growth stage, the bacterial rhizosphere communities would be primarily structured by their soil histories and resemble their cognate bulk soil communities. Indeed, we found that the bacterial communities at the seed and seedling time points from the bulk soil and rhizosphere were more phylogenetically similar than at later time points, or between different soil histories (Figs 1, 2, and 4). Nonetheless, we also observed that within this phylogenetic similarity, there were widespread taxonomic enrichments in the rhizosphere at the seedling growth stage, compared with the bulk soil and root communities (Fig. S9). This suggests that at initial growth stages, the soil history constrains the composition of the bacterial communities, while individual taxa may be attracted to the new rhizosphere from the surrounding soil and enriched. Future work is needed to determine how active the seedling is in this early attraction.

Across the subsequent growth stages, the most striking observation were the widespread community dynamics and taxonomic enrichments in the bacterial rhizosphere communities (Figs 4B and S9). This was entirely absent in the bulk soil communities (Figs 1B and S9) and more limited in the roots (Figs 3 and S9), although growth stages were significant in structuring all three communities (Table 2). The root communities should be primarily shaped by the plant host across growth stages, as we predicted and observed (Table 2). Conversely, the bulk soil communities should largely experience similar abiotic conditions as the rhizosphere, but with minimal influence from the plant host [66]. As we have previously observed, different climate scenarios, such as a dry season versus a non-dry season, can have dramatic effects on bacterial community assembly over a growing season [30]. Based on the temperature and precipitation data collected during our present field experiment, there was little deviation from the 30-year average (Table S1). This would suggest that we should not expect the climate factors to overwhelm any of the several other influences that contribute to the changes which accrue in bacterial bulk soil communities over a growing season. As such, we predicted that these communities would remain largely stable through time, though we actually found that the bulk soil communities were also significantly structured at each time point (Table 2). The significance of the time points structuring the bulk soil communities is likely a reflection of the aging soil.

As the bulk soil ages through time, the bacterial communities will still accumulate changes based on the priority effects established by the soil history, as determined by the soil chemistry, the extant soil macro and micro-communities, climate factors, and the agricultural management practices. The strength of including bulk soil samples in our study is that they allow us to try and untangle previous soil histories from the influence of the host plant. Here, we see that soil history was significant in structuring the bulk soil communities across the growing season, even in the absence of a previous plant host (Table 2, Fig. 1). Since our bulk soil samples were taken from between the seeded rows, it is unlikely that the previous plant hosts influenced the bulk soil communities either, and yet, these communities still show similarities to the rhizosphere communities based on soil history (Fig. 2). Thus, future field experiments should also include unmanaged fallow treatments to further partition the influence of host plants, climate, and management practices, in establishing soil histories, and their longevity.

Unlike the bulk soil, however, we predicted that the bacterial rhizosphere communities would be primarily structured by PSF through time, due to the declining influence of soil history. Therefore, we expected that we expected that by the flower growth stage the rhizosphere communities would be more similar to one another, regardless of their soil history, and more divergent from their cognate bulk soil communities. We did confirm that the rhizosphere communities varied significantly from their bulk soil communities (Figs 2B and S9), and we observed an increase in phylogenetic diversity at later growth stages (Figs 1A and 4A), which both endorses the prediction. It also mirrors the root communities, which were also significantly influenced by growth stages, though never by soil history (Table 2), and also demonstrated increasing variation through time (Fig. 3). In both the bulk soil and rhizosphere communities, we observed increased phylogenetic diversity over time, as older soils can slowly accumulate new community members due to different dispersal, drift, selection, or speciation/diversification events, and the influences of different biotic (e.g. extant soil macro and micro-communities) and abiotic (e.g. soil chemistry, agricultural management practices, and climate) factors [67].

However, rhizosphere communities are also affected by the changing influences of the *B. napus* plant host at each growth stage, while those in the bulk soil are not since they are non-planted controls. Thus, we ought to consider that the trend among the rhizosphere communities may also be due in part to the plant host, unlike in the bulk soil. Similar increases in phylogenetic diversity across growth stage have also been observed in the rhizosphere of other plants, such as rice [68]. Unlike previous findings in the perennial *Brassicaceae Arabidopsis alpina* that showed quite static bacterial communities [69], our data aligned similarly to other annual crops that exhibit dynamic bacterial rhizosphere communities across growth stages [68]. This late growth stage variation observed in all three communities could also be due to the inherent stochasticity of priority effects, where community composition at later growth stages is constrained by earlier stages [31–33]. This could be better tested in the future as high-frequency sampling from multiple hosts of the same genotype may be experimentally valid to detect priority effects [70].

Critically, we did not find that flower growth stage rhizosphere communities were more similar to one another, regardless of their soil histories, as expected (Fig. 2B). In our previous work, we found that diverse *Brassicaceae* hosts at the flower growth stage converged toward phylogenetically similar rhizosphere communities, regardless of their different soil histories [30]. In our current experiment, the plant’s growth stages clearly do become more important in structuring the rhizosphere throughout the growing season. However, additional factors also continue to add diversity and prevent more convergence among the rhizosphere communities. This could further highlight the impact of priority effects in shaping subsequent bacterial communities [31–33]. Including other diverse host plants could better reveal how similar within-host rhizosphere communities are at different growth stages [30].

Alternatively, despite not observing communities from the different soil histories coalesce at the flower growth stage, it is possible that the same *B. napus* host could still assemble rhizosphere communities in different soils that remains functionally similar [71]. At the same time, bacteria can be phylogenetically identical and yet possess different ecological functions [72, 73]. Therefore, functionally explicit sampling—in tandem with the time-series strategy we have advanced here—will be another key approach to unraveling how soil history impacts bacterial rhizosphere communities [74, 75].

### Bacterial diversity was higher with more diverse soil history

Our results further illustrated that bacterial bulk soil and rhizosphere communities with the PBC history consistently had the highest phylogenetic diversity, compared with the monocrop and WC soil histories across all growth stages (Figs 1A and 4A). We can be confident that the increased phylogenetic diversity among the PBC soil communities was due to the different soil histories for two reasons. First, the increase in phylogenetic diversity among PBC plots in the bulk soil communities was present from the first growth stage, before any plant hosts was even present. Second, the increase in phylogenetic diversity in the bulk soil and rhizosphere communities from the PBC plots remained throughout the growing season. Thus, even with the addition of a plant host, the common PBC soil history still impacted the bacterial communities at each growth stage, or concordant sampling time in the case of the bulk soil.

Conversely, we found no difference between the three soil histories in the root communities (Fig. 3), where soil history was not significant to those communities (Table 2). Thus, from our experiment, we can only observe a change in the root communities according to growth stages (Fig. 3). This is consistent with our previous work that also demonstrated that root communities tend to be strongly influenced by the host plant, and weakly impacted by soil history, unless the plant host is stressed, and unable to contribute to PSF [30, 43].

Although we found a clear impact of different soil histories on phylogenetic diversity in the bacterial bulk soil and rhizosphere communities (Table 2, Figs 1A and 4A), it is interesting to note that we only identified a slight change in taxa composition in the bulk soil due to soil history (Fig. 1B), but not the rhizosphere (Fig. 4). Moreover, we did not detect specific ASVs within rhizosphere communities according to their different soil histories. This lack of compositional difference between soil histories might suggest that the different agricultural treatments involved in establishing the previous soil histories were not sufficiently diverse. However, given our previous results using crop rotations, this seems unlikely [30, 43]. Alternatively, the lack of compositional differences between rhizosphere communities despite coming from different soil histories could be evidence for the common host plant structuring similar rhizosphere communities. This would be consistent with other studies that found declining site-specific bacteria over time, and increasing plant-specific bacteria. Including other diverse host plants, similar to our previous experimental design [30, 43], would allow us to better support this conclusion.

## Conclusion

In this experiment, we tested the hypothesis that previously established soil histories would decrease in influencing the structure of *B. napus* bacterial rhizosphere communities over the growing season. We largely confirmed our first and second predictions, which had suggested that the bacterial bulk soil and root communities would be primarily structured by soil history and growth stages, respectively. Our results for rhizosphere communities at the initial seed and seedling stages also confirmed our prediction that these communities would remain similar to their corresponding bulk soil communities and soil histories. We also found that the bacterial bulk soil, rhizosphere, and root communities all diverged more as they aged. However, the rhizosphere communities did not converge in similarity over the growing season, regardless of their soil history, refuting our final prediction. In fact, soil history continued to be influential among the rhizosphere communities across the different growth stages. Furthermore, we show that soil histories established with more plant diversity contribute to more phylogenetically diverse bacterial communities. Therefore, based on our data, our initial hypothesis concerning the decline of soil history influencing the structure of the bacterial rhizosphere communities across the growing season was only partly supported. Instead, we found a strong impact of soil history on the bacterial rhizosphere communities throughout the growing season that stressed the nuanced interactions of rhizosphere microbial community feedback.

Our results highlight the importance of studying microbial communities through time, which has largely been ignored to date [29, 76, 77]. Studying how communities arrive at a given composition is more instructive than just a static snapshot. Here, we found that different soil histories persisted and impacted bacterial diversity throughout the growing season. This suggests that the host plant’s capacity to “re-write” different soil histories may be quite limited as key components that constitute the soil history’s identity remained present and continued to impact the bacterial communities. From the agricultural perspective, persisting soil histories may have important long-term consequences, such as building capacity for more resilient, diverse bacterial communities [22]. This presents exciting future experiments to uncover the transmission components, or memory, of soil history among soil bacterial communities. Given the significant and myriad human-induced changes throughout the biosphere [78], there is a clear need to better account for how historical events may structure rhizosphere microbial communities going forward through time and more broadly influence the mechanisms of community ecology.

## Supplementary Material

Blakney_et_al__2023_ISME_Comms_Supp_Materials_Clean_Copy_ycae019

## Data Availability

Sequencing data and metadata are available at NCBI Bioproject under accession number: PRJNA997731.

## References

[1] Zilber-Rosenberg I , Rosenberg E. Role of microorganisms in the evolution of animals and plants: the hologenome theory of evolution. FEMS Microbiol Re*v* 2008;32:723–35. 10.1111/j.1574-6976.2008.00123.x 18549407

[2] Puginier C , Keller J, Delaux PM. Plant–microbe interactions that have impacted plant terrestrializations. Plant Physio*l* 2022;190:72–84. 10.1093/plphys/kiac258 35642902 PMC9434271

[3] Vandenkoornhuyse P , Quaiser A, Duhamel M et al. The importance of the microbiome of the plant holobiont. New Phyto*l* 2015;206:1196–206. 10.1111/nph.13312 25655016

[4] Richardson AE , Barea JM, McNeill AM et al. Acquisition of phosphorus and nitrogen in the rhizosphere and plant growth promotion by microorganisms. Plant Soi*l* 2009;321:305–39. 10.1007/s11104-009-9895-2

[5] Weidner S , Koller R, Latz E et al. Bacterial diversity amplifies nutrient-based plant–soil feedbacks. Funct Eco*l* 2015;29:1341–9. 10.1111/1365-2435.12445

[6] Yu P , He X, Baer M et al. Plant flavones enrich rhizosphere *Oxalobacteraceae* to improve maize performance under nitrogen deprivation. Nat Plant*s* 2021;7:481–99. 10.1038/s41477-021-00897-y 33833418

[7] Lau JA , Lennon JT. Rapid responses of soil microorganisms improve plant fitness in novel environments. Proc Natl Acad Sci US*A* 2012;109:14058–62. 10.1073/pnas.1202319109 22891306 PMC3435152

[8] Marasco R , Rolli E, Ettoumi B et al. A drought resistance- promoting microbiome is selected by root system under desert farming. PLoS On*e* 2012;7:e48479. 10.1371/journal.pone.0048479 23119032 PMC3485337

[9] Hou S , Thiergart T, Vannier N et al. A microbiota–root– shoot circuit favours *Arabidopsis* growth over defence under suboptimal light. Nat Plant*s* 2021;7:1078–92. 10.1038/s41477-021-00956-4 34226690 PMC8367822

[10] Mendes R , Kruijt M, de Bruijn I et al. Deciphering the rhizosphere microbiome for disease-suppressive bacteria. Scienc*e* 2011;332:1097–100. 10.1126/science.1203980 21551032

[11] Sikes BA , Cottenie K, Klironomos JN. Plant and fungal identity determines pathogen protection of plant roots by arbuscular mycorrhizas. J Eco*l* 2009;97:1274–80. 10.1111/j.1365-2745.2009.01557.x

[12] Chaney L , Baucom RS. The soil microbial community alters patterns of selection on flowering time and fitness-related traits in *Ipomoea purpurea*. Am J Bo*t* 2020;107:186–94. 10.1002/ajb2.1426 32052423 PMC7065020

[13] O'Brien AM , Ginnan NA, Rebolleda-Gómez M et al. Microbial effects on plant phenology and fitness. Am J Bo*t* 2021;108:1824–37. 10.1002/ajb2.1743 34655479

[14] Castrillo G , Teixeira PJPL, Paredes SH et al. Root microbiota drive direct integration of phosphate stress and immunity. Natur*e* 2017;543:513–8. 10.1038/nature21417 28297714 PMC5364063

[15] Lebeis SL , Paredes SH, Lundberg DS et al. Salicylic acid modulates colonization of the root microbiome by specific bacterial taxa. Scienc*e* 2015;349:860–4. 10.1126/science.aaa8764 26184915

[16] Korenblum E , Dong Y, Szymanski J et al. Rhizosphere microbiome mediates systemic root metabolite exudation by root-to-root signaling. Proc Natl Acad Sci US*A* 2020;117:3874–83. 10.1073/pnas.1912130117 32015118 PMC7035606

[17] Kawasaki A , Dennis PG, Forstner C et al. Manipulating exudate composition from root apices shapes the microbiome throughout the root system. Plant Physio*l* 2021;187:2279–95. 10.1093/plphys/kiab337 34618027 PMC8644255

[18] Grady KL , Sorensen JW, Stopnisek N et al. Assembly and seasonality of core phyllosphere microbiota on perennial biofuel crops. Nat Comm*s* 2019;10:4135–10. 10.1038/s41467-019-11974-4 PMC6742659 31515535

[19] Hu L , Wu Z, Robert CAM et al. Soil chemistry determines whether defensive plant secondary metabolites promote or suppress herbivore growth. Proc Natl Acad Sci US*A* 2021;118:e2109602118. 10.1073/pnas.2109602118 34675080 PMC8639379

[20] Song Y , Wilson AJ, Zhang XC et al. FERONIA restricts *pseudomonas* in the rhizosphere microbiome via regulation of reactive oxygen species. Nat Plant*s* 2021;7:644–54. 10.1038/s41477-021-00914-0 33972713

[21] Hwang SF , Ahmed HU, Turnbull GD et al. Effect of seeding date and depth, seed size and fungicide treatment on *Fusarium* and *Pythium* seedling blight of canola. Can J Plant Sc*i* 2015;95:293–301. 10.4141/cjps-2014-268

[22] Yang T , Lupwayi N, St-Arnaud M et al. Anthropogenic drivers of soil microbial communities and impacts on soil biological functions in agroecosystems. Global Ecol Conser*v* 2021;27:e01521. 10.1016/j.gecco.2021.e01521

[23] Liu B , Arlotti D, Huyghebaert B et al. Disentangling the impact of contrasting agricultural management practices on soil microbial communities – importance of rare bacterial community members. Soil Biol Bioche*m* 2022;166:108573. 10.1016/j.soilbio.2022.108573

[24] Bever JD , Dickie IA, Facelli E et al. Rooting theories of plant community ecology in microbial interactions. Trends Ecol Evo*l* 2010;25:468–78. 10.1016/j.tree.2010.05.004 20557974 PMC2921684

[25] Kaisermann A , de Vries FT, Griffiths RI et al. Legacy effects of drought on plant–soil feedbacks and plant–plant interactions. New Phyto*l* 2017;215:1413–24. 10.1111/nph.14661 28621813

[26] Berendsen RL , Vismans G, Yu K et al. Disease- induced assemblage of a plant-beneficial bacterial consortium. ISME *J* 2018;12:1496–507. 10.1038/s41396-018-0093-1 29520025 PMC5956071

[27] Fitzpatrick CR , Copeland J, Wang PW et al. Assembly and ecological function of the root microbiome across angiosperm plant species. Proc Natl Acad Sci US*A* 2018;115:E1157–65. 10.1073/pnas.1717617115 29358405 PMC5819437

[28] Bakker PAHM , Pieterse CMJ, de Jonge R et al. The soil-borne legacy. Cel*l* 2021;172:1178–80 10.1016/j.cell.2018.02.024 29522740

[29] Hannula SE , Heinen R, Huberty M et al. Persistence of plant-mediated microbial soil legacy effects in soil and inside roots. Nature Comm*s* 2021;12:5686. 10.1038/s41467-021-25971-z PMC8478921 34584090

[30] Blakney AJC , Bainard LD, St-Arnaud M et al. *Brassicaceae* host plants mask the feedback from the previous year’s soil history on bacterial communities, except when they experience drought. Environ Microbio*l* 2022;24:3529–48. 10.1111/1462-2920.16046 35590462

[31] Mseisner A , Snoek BL, Nesme J et al. Soil microbial legacies differ following drying-rewetting and freezing-thawing cycles. ISME *J* 2021;15:1207–21. 10.1038/s41396-020-00844-3 33408369 PMC8115648

[32] Chase AB , Weihe C, Martiny JBH. Adaptive differentiation and rapid evolution of a soil bacterium along a climate gradient. Proc Nat Acad Sci USA*s* 2021;118:e2101254118. 10.1073/pnas.2101254118 PMC8106337 33906949

[33] Debray R , Herbert RA, Jaffe AL et al. Priority effects in microbiome assembly. Nat Rev Microbio*l* 2022;20:109–21. 10.1038/s41579-021-00604-w 34453137

[34] Links MG , Demeke T, Gräfenhan T et al. Simultaneous profiling of seed-associated bacteria and fungi reveals antagonistic interactions between microorganisms within a shared epiphytic microbiome on *Triticum* and *brassica* seeds. New Phyto*l* 2014;202:542–53. 10.1111/nph.12693 24444052 PMC4235306

[35] Nelson EB , Simoneau P, Barret M et al. Editorial special issue: the soil, the seed, the microbes and the plant. Plant Soi*l* 2018;422:1–5. 10.1007/s11104-018-3576-y

[36] Rezki S , Campion C, Simoneau P et al. Assembly of seed-associated microbial communities within and across successive plant generations. Plant Soi*l* 2018;422:67–79. 10.1007/s11104-017-3451-2

[37] Eldridge DJ , Travers SK, Val J et al. Experimental evidence of strong relationships between soil microbial communities and plant germination. J Eco*l* 2021;109:2488–98. 10.1111/1365-2745.13660

[38] Shao J , Miao Y, Liu K et al. Rhizosphere microbiome assembly involves seed-borne bacteria in compensatory phosphate solubilization. Soil Biol Bioche*m* 2021;159:108273–8. 10.1016/j.soilbio.2021.108273

[39] Shade A , McManus PS, Handelsman J. Unexpected diversity during community succession in the apple flower microbiome. MBi*o* 2013;4:e00602–12. 10.1128/mBio.00602-12 23443006 PMC3585449

[40] Wagner MR , Lundberg DS, Coleman-Derr D et al. Natural soil microbes alter flowering phenology and the intensity of selection on flowering time in a wild Arabidopsis relative. Ecol Let*t* 2014;17:717–26. 10.1111/ele.12276 24698177 PMC4048358

[41] Cui Z , Huntley RB, Zeng Q et al. Temporal and spatial dynamics in the apple flower microbiome in the presence of the phytopathogen *Erwinia amylovora*. ISME *J* 2021;15:318–29. 10.1038/s41396-020-00784-y 33024293 PMC7853089

[42] Walsh CM , Becker-Uncapher I, Carlson M et al. Variable influences of soil and seed-associated bacterial communities on the assembly of seedling microbiomes. ISME *J* 2021;15:2748–62. 10.1038/s41396-021-00967-1 33782567 PMC8397733

[43] Blakney AJC , Bainard LD, St-Arnaud M et al. Soil chemistry and soil history significantly structure oomycete communities in *Brassicaceae* crop rotations. Appl Environ Microbio*l* 2023;89:1–22. 10.1128/aem.01314-22 PMC9888183 36629416

[44] Chung YA . The temporal and spatial dimensions of plant–soil feedbacks. New Phyto*l* 2023;237:2012–9. 10.1111/nph.18719 36604846

[45] De Long JR , Heinen R, Heinze J et al. Plant-soil feedback: incorporating untested influential drivers and reconciling terminology. Plant Soi*l* 2023;485:7–43. 10.1007/s11104-023-05908-9

[46] De Vrieze J , De Mulder T, Matassa S et al. Stochasticity in microbiology: managing unpredictability to reach the sustainable development goals. Microb Biotechno*l* 2020;13:829–43. 10.1111/1751-7915.13575 32311222 PMC7264747

[47] Revillini D , Gehring CA, Johnson NC. The role of locally adapted mycorrhizas and rhizobacteria in plant–soil feedback systems. Funct Eco*l* 2016;30:1086–98. 10.1111/1365-2435.12668

[48] Harker KN , O’Donovan JT, Turkington TK et al. Canola rotation frequency impacts canola yield and associated pest species. Can J Plant Sc*i* 2015;95:9–20. 10.4141/cjps-2014-289

[49] Canola Council of Canada . Canola Canola Encyclopedia: Canola Growth Stage*s*. Manitoba, Canada: Winnipeg, 2017

[50] Delavaux CS , Bever JD, Karppinen EM et al. Keeping it cool: soil sample cold pack storage and DNA shipment up to 1 month does not impact metabarcoding results. Ecol Evo*l* 2020;10:4652–64. 10.1002/ece3.6219 32551050 PMC7297747

[51] Lay CY , Bell TH, Hamel C et al. Canola root– associated microbiomes in the Canadian prairies. Front Microbio*l* 2018;9:1–19. 10.3389/fmicb.2018.01188 29937756 PMC6002653

[52] Bell TH , Stefani FOP, Abram K et al. A diverse soil microbiome degrades more crude oil than specialized bacterial assemblages obtained in culture. Appl Environ Microbio*l* 2016;82:5530–41. 10.1128/AEM.01327-16 27371586 PMC5007768

[53] Callahan BJ , McMurdie PJ, Rosen MJ et al. DADA2: high-resolution sample inference from Illumina amplicon data. Nat Method*s* 2016;13:581–3. 10.1038/nmeth.3869 27214047 PMC4927377

[54] Azarbad H , Constanta P, Giard-Laliberté C et al. Water stress history and wheat genotype modulate rhizosphere microbial response to drought. Soil Biol Bioche*m* 2018;126:228–36. 10.1016/j.soilbio.2018.08.017

[55] Kembel SW , Cowan PD, Helmus MR et al. Picante: R tools for integrating phylogenies and ecology. Bioinf*o* 2010;26:1463–4 10.1093/bioinformatics/btq166 20395285

[56] Foster Z , Sharpton T, Grünwald N. Metacoder: an R package for visualization and manipulation of community taxonomic diversity data. PLoS Comput Bio*l* 2017;13:s1–15. 10.1371/journal.pcbi.1005404 PMC5340466 28222096

[57] Legendre P , Legendre L. Numerical Ecolog*y*. The Netherlands: Elsevier, 2012

[58] Oksanen J , Blanchet FG, Friendly M et al. Vegan: Community Ecology Package. 2020, R package version 2.5-7.

[59] Lozupone CA , Knight R. UniFrac: a new phylogenetic method for comparing microbial communities. Appl Environ Microbio*l* 2005;71:8228–35. 10.1128/AEM.71.12.8228-8235.2005 16332807 PMC1317376

[60] Lozupone CA , Hamady M, Kelley ST et al. Quantitative and qualitative diversity measures lead to different insights into factors that structure microbial communities. Appl Environ Microbio*l* 2007;73:1576–85. 10.1128/AEM.01996-06 17220268 PMC1828774

[61] McMurdie P , Holmes S. Phyloseq: an R package for reproducible interactive analysis and graphics of microbiome census data. PLoS On*e* 2013;8:e61217. 10.1371/journal.pone.0061217 23630581 PMC3632530

[62] Carteron A , Beigas M, Joly S et al. Temperate forests dominated by arbuscular or ectomycorrhizal fungi are characterized by strong shifts from saprotrophic to mycorrhizal fungi with increasing soil depth. Microb Eco*l* 2021;82:377–90. 10.1007/s00248-020-01540-7 32556393

[63] Callahan BJ , McMurdie PJ, Holmes SP. Exact sequence variants should replace operational taxonomic units in marker-gene data analysis. ISME *J* 2017;11:2639–43. 10.1038/ismej.2017.119 28731476 PMC5702726

[64] Martinović T , Odriozola I, Mašínová T et al. Temporal turnover of the soil microbiome composition is guild-specific. Ecol Let*t* 2021;24:2726–38. 10.1111/ele.13896 34595822

[65] Gu Y , Banerjee S, Dini-Andreote F et al. Small changes in rhizosphere microbiome composition predict disease outcomes earlier than pathogen density variations. ISME *J* 2022;16:2448–56. 10.1038/s41396-022-01290-z 35869387 PMC9478146

[66] Vieira S , Sikorski J, Dietz S et al. Drivers of the composition of active rhizosphere bacterial communities in temperate grasslands. ISME *J* 2020;14:463–75. 10.1038/s41396-019-0543-4 31659233 PMC6976627

[67] Nemergut DR , Schmidt SK, Fukami T et al. Patterns and processes of microbial community assembly. Microbiol Mol Biol Re*v* 2013;77:342–56. 10.1128/MMBR.00051-12 24006468 PMC3811611

[68] Edwards JA , Santos-Medellín CM, Liechty ZS et al. Compositional shifts in root-associated bacterial and archaeal microbiota track the plant life cycle in field-grown rice. PLoS Bio*l* 2018;16:e2003862. 10.1371/journal.pbio.2003862 29474469 PMC5841827

[69] Dombrowski N , Schlaeppi K, Agler M et al. Root microbiota dynamics of perennial *Arabis alpina* are dependent on soil residence time but independent of flowering time. ISME *J* 2017;11:43–55. 10.1038/ismej.2016.109 27482927 PMC5097464

[70] Mamet SD , Helgason BL, Lamb EG et al. Phenology-dependent root bacteria enhance yield of *Brassica napus*. Soil Biol Bioche*m* 2022;166:108468. 10.1016/j.soilbio.2021.108468

[71] Yang Y . Emerging patterns of microbial functional traits. Trends Microbio*l* 2021;29:874–82. 10.1016/j.tim.2021.04.004 34030967

[72] Blakney AJC , Patten CL. A plant growth-promoting pseudomonad is closely related to the *Pseudomonas syringae* complex of plant pathogens. FEMS Microbiol Eco*l* 2011;77:546–57. 10.1111/j.1574-6941.2011.01136.x 21609343

[73] Gundersen MS , Morelan IA, Andersen T et al. The effect of periodic disturbances and carrying capacity on the significance of selection and drift in complex bacterial communities. ISME Comm*s* 2021;1:53–62. 10.1038/s43705-021-00058-4 PMC9723678 37938282

[74] King WL , Yates CF, Cao L et al. Functionally discrete fine roots differ in microbial assembly, microbial functional potential, and produced metabolites. Plant Cell Enviro*n* 2023;46:3919–32. 10.1111/pce.14705 37675977

[75] Fleishman SM , Eissenstat DM, Bell TH et al. Functionally-explicit sampling can answer key questions about the specificigy of plant–microbe interactions. Environ Microbiom*e* 2022;17:51. 10.1186/s40793-022-00445-x 36221138 PMC9555203

[76] Hannula SE , Kielak AM, Steinauer K et al. Time after time: temporal variation in the effects of grass and forb species on soil bacterial and fungal communities. MBi*o* 2019;10:e02635–19. 10.1128/mBio.02635-19 31848279 PMC6918080

[77] Chung YA . The temporal and spatial dimensions of plant–soil feedbacks. New Phyto*l* 2023;237:2012–9. 10.1111/nph.18719 36604846

[78] IPCC . In: Masson-Delmotte V., Zhai P., Pirani A. et al. (eds.), *Climate Change 2021: The Physical Science Basis.* Contribution of Working Group I to the Sixth Assessment Report of the Intergovernmental Panel on Climate Change. Cambridge, United Kingdom and New York, NY, USA: Cambridge University Press, 2021, In press.

